# TEP or TAPP: who, when, and how?

**DOI:** 10.3389/fsurg.2024.1352196

**Published:** 2024-07-15

**Authors:** Angelo Iossa, Giovanni Traumueller Tamagnini, Francesco De Angelis, Alessandra Micalizzi, Giulio Lelli, Giuseppe Cavallaro

**Affiliations:** Department of Medical-Surgical Sciences and Biotechnologies, Faculty of Pharmacy and Medicine, “La Sapienza” University of Rome-Polo Pontino, Bariatric Centre of Excellence SICOB, Latina, Italy

**Keywords:** laparoendoscopic inguinal hernia surgery, TAPP, TEP, review of literature, laparoscopic groin hernia repair

## Abstract

Groin hernia repair is the most common procedure performed by general surgeons. The open mesh technique generally represents the main technique for an inguinal repair, but a different approach is often required. Laparoscopy was found to be the answer to minimizing the impact of the preperitoneal open techniques described by Nyhus and Stoppa. The introduction of the totally extraperitoneal hernia repair (TEP) and transabdominal preperitoneal repair (TAPP) in the early 1990s started a new chapter in groin hernia surgery. The minimally invasive techniques vs. open mesh, and then one against the other, soon became a hot topic among abdominal wall surgeons. With time, the number of procedures and indications increased and are still increasing. This review aims to provide an overview of the two main laparoscopic techniques for groin hernia repair, answering the following questions: Who should perform them? What is the learning curve required to minimize complications and optimize operative time? When is a minimally invasive approach indicated, and which one (both in elective and in emergency setting)? How are they performed? The standard techniques have been described in detail, and personal observations from an abdominal wall surgery referral center were added. The main reviews from the early 2000s up to date, which compared the techniques, were analyzed, and the results reported, confirming the comparable safety and efficacy of both these techniques.

## Introduction

The laparoscopic approach for the treatment of groin hernias was introduced to the international surgical community in the early 1990s as a minimally invasive version of the well-known preperitoneal open repair technique described by Nyhus and Stoppa. The laparoscopic approach to hernia repair was initially described by Ger et al. (1). In 1992, Arregui et al. presented a preliminary report on the transabdominal pre-peritoneal repair (TAPP) in 52 patients (2), while Dulucq, Mckernan, Phillips, and Ferzli (3–6) recommended the totally extra-pre-peritoneal repair (TEP), which avoided the violation of the peritoneal cavity. The most common modern laparoscopic techniques for inguinal hernia repair are transabdominal preperitoneal (TAPP) repair and totally extraperitoneal (TEP) repair. TAPP requires access to the peritoneal cavity with the placement of a mesh through a peritoneal incision. This mesh is placed in the preperitoneal space covering all potential hernia sites in the inguinal region. The peritoneum is then closed above the mesh. TEP is different, being a totally extraperitoneal procedure. The mesh is used to cover the hernia from outside the peritoneum. This approach is more difficult than TAPP but may lessen the risks of damage to the internal organs and of adhesion formation leading to intestinal obstruction, which has been linked to TAPP.

Laparoscopic repair is technically more difficult than open repair, and there is evidence of a “learning curve” in its performance, requiring advanced surgical skills such as suturing and bimanual dissection, together with a pre-peritoneal space creation, which is not considered a common, standard approach for general surgeons. Likely, some of the higher rates of potentially serious complications reported for laparoscopic repair are associated with learning effects, particularly for the more complex TEP repair. The comparison between TAPP and TEP soon became a hot topic, evaluating the pros and cons, weaknesses, and strengths of the two both in the elective and emergency contexts.

Worldwide, more than 20 million patients undergo groin hernia repair every year (7). A nationwide US analysis of laparoscopic vs. open inguinal hernia repair during the period 2009–2015, including a total of 41,937 patients, reports that 87.2% underwent open repair, while 12.8% underwent laparoscopic repair. A nationwide analysis of laparoscopic groin hernia repair in Italy from 2015 to 2020 showed that 33,925 procedures were performed with a mean annual change of 8.60% from 2015 to 2019, accounting for 3.56% of all hernia repair procedures in 2015 and 5.98% in 2020, with almost similar percentages of bilateral and unilateral hernias. In the same analysis, the conversion rate to open surgery decreased from 2015 to 2019 with a mean annual change of −1.14%, although not significantly different (8). Compared to the standard open approach (Lichtenstein), TAPP and TEP repairs seem associated with significantly reduced early postoperative pain, return to work/activities, chronic pain, hematoma, and wound infection (9).

In the present paper, we want to report the updated indications to perform TAPP or TEP and describe technical details about the two operations with a discussion on an updated comparison between the two procedures.

## Who and when?

Regarding the question of **who**, we must state that the learning curve and adequate technical details remain the key to reducing complications, conversion, and operative time.

The European Hernia Society guidelines in 2009 suggest that the learning curve for laparoscopic repairs is between 50 and 100 procedures (10). The EndoHerniaSociety (IEHS) guidelines in 2011 indicated “expert,” surgeons in a range between 30 and 100 procedures (11). The international guidelines by the HerniaSurge group in 2018 confirmed a learning curve for laparoscopic inguinal hernia repair between 50 and 100 procedures (12). In 2023, Sivakumar et al. published a systematic review, a meta-analysis, and a meta-regression of the learning curve of laparoscopic inguinal hernia repair, reporting a median number of cases required to overcome the learning curve for laparoscopic inguinal hernia of 35.7 consisting of 34.15 (range, 14–80) and 37.5 (range, 13–75) procedures for TEP/single incision (SIL) TEP and TAPP, respectively. Mixed-effects Poisson regression demonstrated a non-linear trend in the number of cases required to surmount the learning curve for laparoscopic inguinal hernia repairs from 1995 to 2020. This model found a significant decrease of 2.7% year-on-year in the number of cases for the learning curve threshold to be achieved (95% CI −4.1% to −1.2%). The predicted number of cases needed to surmount the learning curve in 2020 was 32.5 (*p* < 0.01). When the analysis was performed independently for each operative approach, the fitted model demonstrated a threshold of 34.4 cases for TEP/SILTEP (*p* < 0.01) and 22.7 cases for TAPP (*p* = 0.017) in 2020 (13).

Regarding **when** to perform, the international and European guidelines consider both TAPP and TEP as a single entity, indicating that a minimally invasive approach is comparable to Lichtenstein repair in unilateral hernia in male patients. In terms of the recurrence rate, it provides a lower risk of postoperative inguinal pain and hematoma development despite carrying a higher risk of postoperative seroma formation and higher costs per procedure. In unilateral groin hernia of the female and bilateral groin hernias, the laparoscopic approach is strongly recommended due to a complete control of the myopectineal orifice and, consequently, of the femoral region. It can also minimize invasivity with the use of the same three accesses for bilateral defects. Moreover, in the case of recurrence following a previous open hernia repair, the laparoscopic approach is advised (12). In all cases, the recommendations are based on the availability of trained and expert surgeons due to the relatively long learning curve for minimally invasive techniques.

The IEHS 2011 (11) and HerniaSurge 2018 (12) guidelines particularly supported the need to choose a different way to treat recurrent hernias (after anterior repair) and bilateral disease at the same time if patients are fit for general surgery and did not report specific contraindications. There are some relative contraindications, including large inguinoscrotal hernias, which should not be attempted early in the learning curve as they can be quite difficult operations, and patients on anticoagulation, secondary to the difficulty with dealing with postoperative bleeding in the retroperitoneal space compared to dealing with bleeding after open surgery. Similarly, a history of pelvic surgery should be considered a factor affecting the conversion rate and should be performed only by expert surgeons. Regarding these relative largely recognized contraindications in a retrospective study on 142,052 hernia repairs, of which 21,441 (15%) were on antiplatelet and anticoagulant therapy, the authors reported that compared with the open approach, the rates of 30-day postoperative hematoma, transfusions, stroke, myocardial infarction, deep venous thrombosis, pulmonary embolism, readmission, and emergency department visits were similar between the two operative approaches (14), underlying that, at present, the relative contraindications are constantly surmounted by expertise and knowledge.

## Emergency setting

In the case of incarcerated/strangulated groin hernias, based on the HerniaSurge guidelines (12), laparoscopic techniques with mesh repair are recommended in the case of a clean and clean/contaminated surgical field as they allow direct inspection of the femoral orifice and, in the case of intraperitoneal access, a surgical exploration of the herniated viscera, which is advised in case of doubt regarding bowel viability. In any case in the emergency context, there is a lack of strong evidence. TAPP represents the best available minimally invasive option for inguinal hernia treatment in an emergency, despite TEP gaining popularity mainly in incarcerated femoral hernias. The World Society of Emergency Surgery (WSES) guidelines in 2017 (15) recommend, in cases of an incarcerated hernia, laparoscopy as a first step of the procedure to assess bowel viability (Grade 2B) and perform the laparoscopic repair only in cases without a need for bowel resection; otherwise, the open approach is considered the best choice (Grade 2C). The study did not take into account the large literature reporting good results in the emergency setting. Zanoni et al. (16) reported the single-center experience on 47 patients submitted to emergent treatment for complicated inguinal hernia with a conversion rate of 4% and no impact on postoperative complications and recurrence rate (0% at 4 years FU). This year, Sartori et al. (17) published a systematic review and a meta-analysis, including 15 articles and 433 patients. A total of 388 patients (75.3%) underwent TAPP, while 103 patients (22.9%) underwent TEP. Herniated structures were resected in 48 cases. Intraoperative complications and conversion occurred in 4 (range 0–1) and 10 (range 0–3) patients, respectively. The mean operative time and hospital stay ranged between 50 and 147 min and 2 and 7 days, respectively. Postoperative complications ranged between 1 and 19. Intraoperative complications and conversion occurred in one (0.6%) and five (2.1%) patients (*p* = 0.4077), concluding that laparoscopy is a safe and feasible approach for the treatment of acute incarcerated groin hernia. Based on the 2023 updated international HerniaSurge guidelines on groin hernia management when approaching an acutely irreducible groin hernia, it is suggested to use diagnostic laparoscopy if expertise and resources are available, and the patient's conditions allow it (weak recommendation). A laparoscopic hernia repair can be attempted if expertise is available (weak recommendation) without specifying what technique between TAPP and TEP has to be preferred (18).

## How?

### TAPP surgical technique

With the patient lying supine, the optical trocar (T1, 11–12 mm) is inserted in a supraumbilical position via open laparoscopy. After lower abdomen inspection, T2 and T3 are positioned on the transverse umbilical line 6–8 cm lateral to T1 under observation and carefully avoiding the inferior epigastric vessels (19). The preparation of a large peritoneal flap is then necessary for a correct exposition of the whole myopectineal orifice and a correct mesh positioning. Guided by old anatomic concepts (20), reported in IEHS guidelines (11), and recently methodologically standardized by Furtado et al. (21) and Claus et al. (22) with “10 golden rules,” the deep inguinal ring is identified through the parietal peritoneum at the center of the “inverted y,” formed superiorly by the inferior epigastric vessels (IEVs) and inferiorly by the vas deferens (or round ligament in female patient) medially and laterally by the testicular vessels (only in male patients). The flap must include all these structures; hence, a transverse incision is performed between a point located 2 cm lower and medial to the superior anterior iliac spine and the lateral umbilical ligament. Dissection is carried out (Step 1) by mobilizing the flap in the space of Bogros, and the epigastric vessels are exposed. The dissection is then conducted medially until Cooper's ligament is identified, as well as part of the femoral orifice. Laterally (Step 2) to the IEVs, the dissection is carried on, inferiorly exposing the iliac fascia, the ileopubic tract (IT), and the ileopsoas fascia. Depending on the defect, the hernia sac is then dissected from the transversalis fascia on Hasselbach's triangle or from the spermatic cord and its structures (round ligament in women). Preserving the round ligament of the uterus is advised by some authors (23), although there is no superiority between preserving and transecting it (24). The transection of the round ligament 1 cm proximal to the deep ring facilitates the dissection but may lead to a worsening pelvic visceral stability in elderly patients. A careful parietalization (Step 3) of the male structures is then mandatory for a correct mesh positioning, especially in the lower limit, to avoid rolling of the mesh, which is a major risk factor for recurrence. The dissection is sufficient as the crossing of the external iliac vein by the vas deferens is exposed and aligned to the iliopsoas fascia. After mesh placement, closure of the peritoneal flap is then performed using a barbed 2/0 suture.

#### Personal modification/considerations

**Figure 1:**
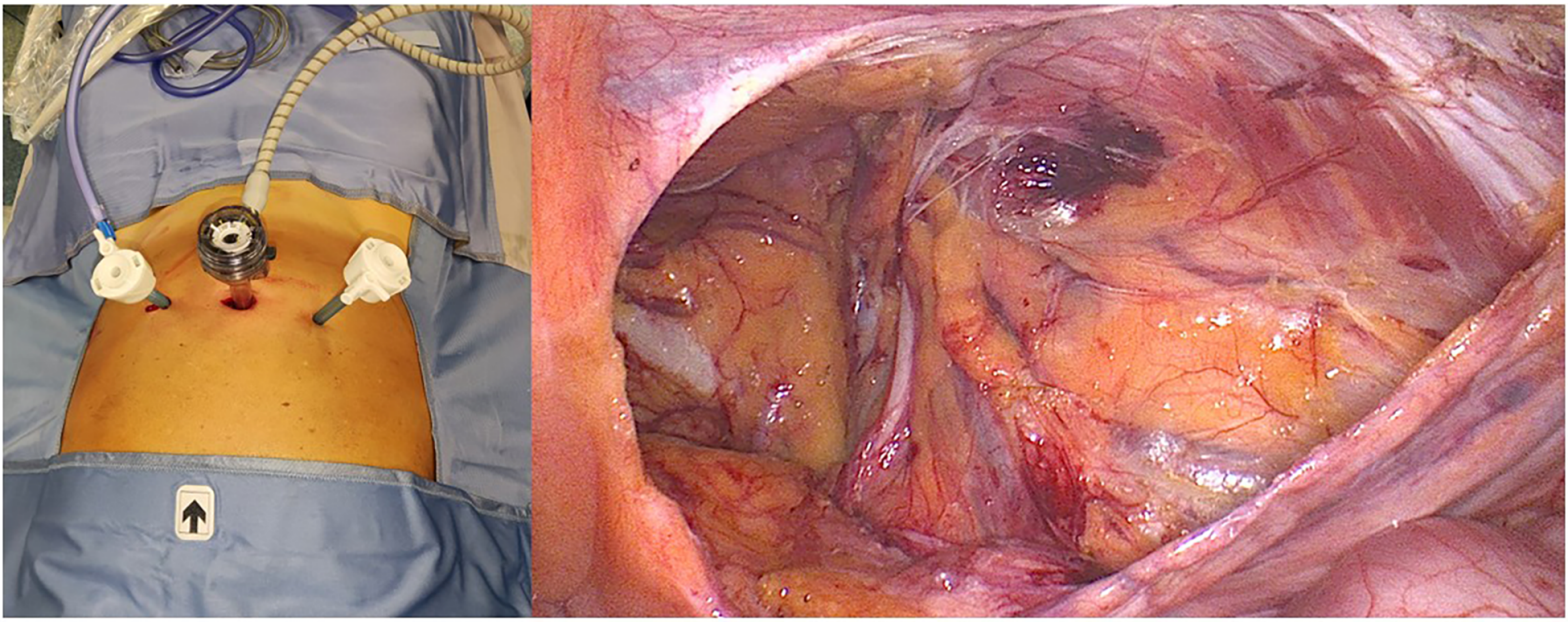
TAPP trocar disposition and inguinal dissection view before the mesh placement.

In our department, we routinely used a 10 mm optical trocar and two 5 mm operative trocars. Independent from the umbilical scar (considered obese or operated patients), we made a 2 cm transverse incision on the midline based on the cutaneous projection of the transverse umbilical line (a line that passes through the abdomen at the middle distance between the iliac crest and the rib inferior margin) for first the trocar (optical T1) placement at least 15 cm inferiorly to the xiphoid bone. All equipment (suture, gauze, and mesh) were placed intrabdominally blind through the optical trocar. Figure 1 shows presents the trocar placement and laparoscopic view before the mesh placement. Mesh fixation is avoided, except in very large EHS-classified M3 hernias, to reduce urinary retention, mean operative time, and postoperative pain at 24 h and 6 months without affecting the recurrence rate (25).

### TEP surgical technique

#### Patient lying supine

Historically, the creation of a pre-peritoneal space and the trocar placement for TEP require the placement of three trocars in the lower midline, that is, one Hasson and two 5 mm trocars. A 15 mm curvilinear infra umbilical incision is made and carried down sharply to the level of the fascia. The anterior rectus sheath is incised transversely off the midline to expose the rectus abdominis muscle. The rectus abdominis muscle is swept, laterally exposing the posterior rectus sheath. The pre-peritoneal space can be created by finger dissection and camera dissection through the Hasson trocar or with the use of a balloon space maker, which is expensive, but a faster choice. A 10 mm 30° laparoscope is routinely used, helping the insertion of two operative 5 mm trocars along the lower midline at the distance of at least two fingers between each other. For hernia dissection and steps, Ferzli's “seven rules” (26) still represent the better way to replicate the procedure and perform a safe approach.

Ferzli's seven steps of TEP:

1. Identify the pubic symphysis in the midline.
2. Bluntly dissect Cooper's ligament bilaterally. This will open up the space of Retzius.
3. Identify Hesselbach's triangle and the three potential sites of herniation related to it (direct, femoral, and obturator).
4. Identify and elevate the epigastric vessels.
5. Bluntly develop the space of Bogros to the level of the ASIS.
6. Dissection of cord structures or round ligaments in females (in accordance with what is declared in the TAPP session)
7. Placement of mesh

#### Personal modification

**Figure 2:**
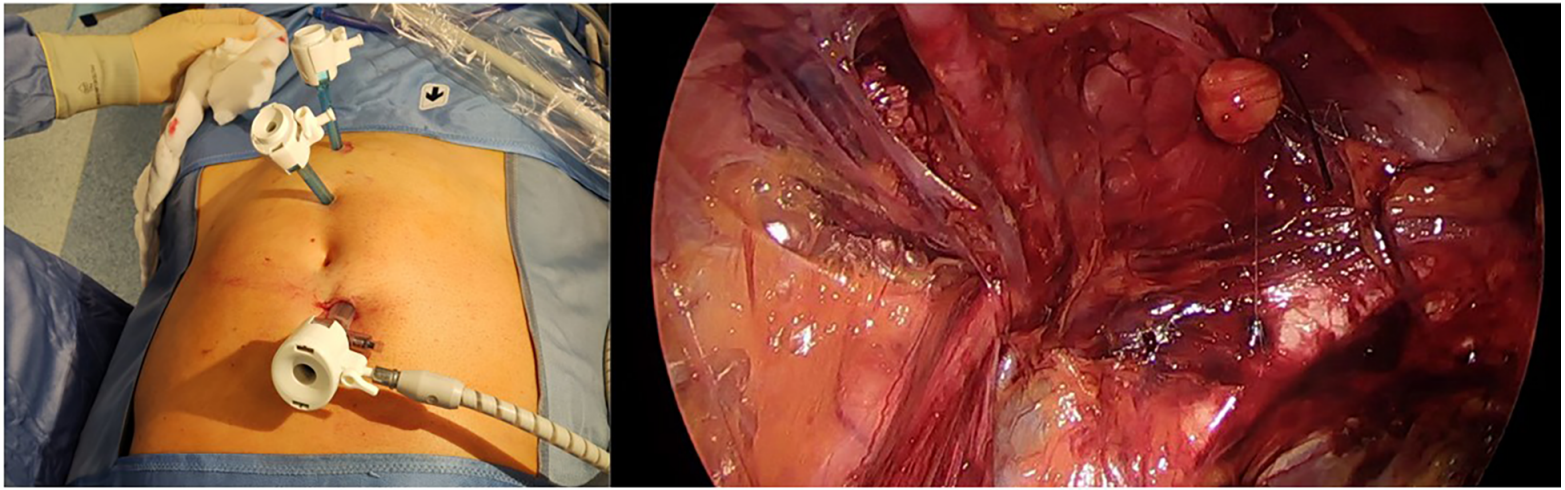
TEP trocar disposition and inguinal dissection view before the mesh placement.

In our department, in the last 5 years, we have routinely changed the position of the first incision. We made a 3 cm transverse incision 2 cm above the umbilical on the cutaneous projection of the transverse umbilical line shifted more on the patient's right side. A transverse incision of the anterior rectus sheet is then made, and the muscle fibers moved laterally exposing the posterior fascia. The following steps are made as for the classic method but with a higher camera port as for the E-TEP procedure, as it allows a larger working space compared to the standard approach, avoids trocar conflicts, and provides easy maneuvers with the tip of the optical trocar placed just above the umbilical scar far more than 5 cm from the first 5 mm trocar on the lower midline. Figure 2 shows our trocar placement and laparoscopic view before the mesh placement. Mesh fixation follows the same TAPP indications.

**Figure 3:**
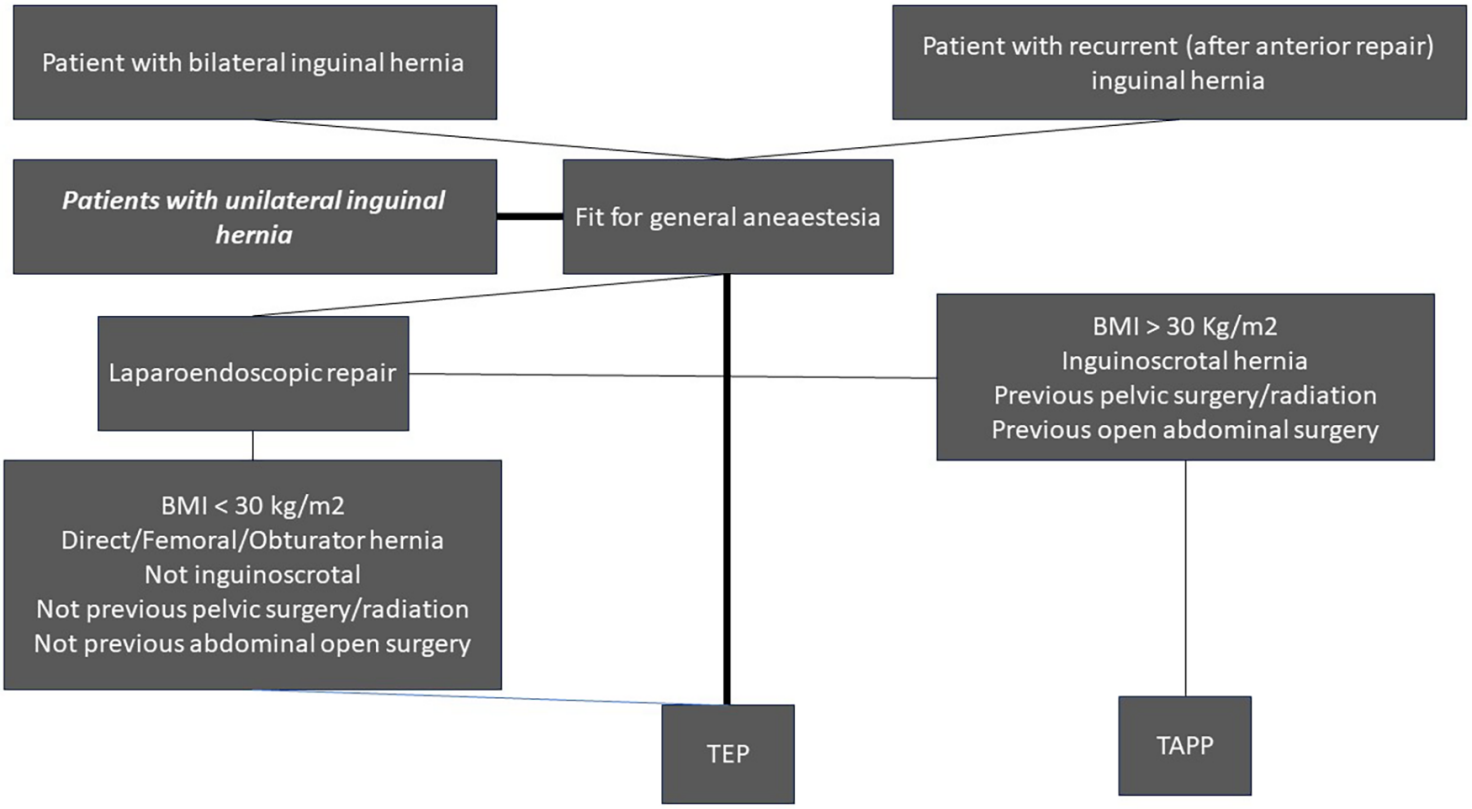
Decisional flowchart on elective groin hernia management.

A personal decisional flowchart on elective groin hernia repair is illustrated in Figure 3.

## Discussion

The comparison between TEP and TAPP almost starts with the first description of the two techniques. With time, it has become a hot topic in the abdominal wall surgical community. Many systematic reviews were published in the last 20 years, even with an initial scarcity of randomized control trials specifically comparing TEP and TAPP. In 2005, a systematic review by Wake et al. (27) included a total of 11,651 patients who underwent the TAPP repair and were compared to a total of 7,043 patients who underwent the TEP repair and gathered from non-randomized studies. The review analyzed the incidence of potentially serious adverse events comparing TAPP vs. TEP with respectively 29 (0.25%) vs. 26 (0.37%) vascular injuries, 54 (0.46%) vs. 12 (0.17%) visceral injuries, 8 (0.07%) vs. 1 (0.01%) deep/mesh infections, 39 (0.33%) vs. 2 (0.03%) port site hernias, 8 (0.07%) vs. 27 (0.38%) conversions. Only one randomized trial (RCT) met the inclusion criteria of the study, which was a clinical diagnosis of groin hernia with indication to surgical treatment, either unilateral or bilateral, in adult patients who underwent either TAPP or TEP repair. The trial compared 28 patients treated with the TAPP technique to 24 patients treated with the TEP technique. The outcomes evaluated were as follows: operation time (mean/SD), 46.0 min (2, 9) for TAPP and 52.3 min (13.9) for TEPP; intraoperative complications (none in both groups); hematoma formation 1/28 vs. 0/24; time to return to usual activities (days); time to return to usual activities (days) (mean/SEM): walking 8.6 (1.4) vs. 8.5 (1.3), driving a car 10.1(1.4) vs. 12.4 (1.7), sexual intercourse 17.7 (2.7) vs. 18.9 (2.6), and sports 35.5 (4.9) vs. 35.2 (4.6); time to return to work (weeks) (mean/SEM) 4.9 (0.7) vs. 4.6 (0.6); length of hospital stay (mean/SD) 3.7 (1.4) vs. 4.4 (0.9), which was the only outcome with a statistically significant difference (*p* < 0.05); and recurrence at 3 months 1/28 vs. 0/24.

The review highlighted that vascular injuries and deep/mesh infections were very rare, and there was no obvious difference between the groups due to the small numbers.

In 2012, Bracale et al. (28) published a systematic review with a network meta-analysis comparing TEP and TAPP. It included only RCTs and indirectly compared TAPP to TEP through a network meta-analysis of studies comparing TAPP to either open hernia repair (OHR) or TEP to OHR due to the scarcity of RCTs directly comparing the two laparoscopic techniques. Seventeen studies were included for a total of 1,209 patients in the TEP group and 395 in the TAPP group. The variables evaluated were operative time, postoperative pain (VAS), hospital stay (days) with TEP associated with a significantly shorter hospital stay than TAPP: −0.31 days (0.082–0.53; *p* < 0.01) (this was the only outcome significantly different from the network, although in part due to a non-significant pooled outcome obtained by comparing TAPP to OHR), time to return to work (days), postoperative complications, and recurrencies. The only outcome significantly different from the network was hospital stay, with TEP providing a shorter hospitalization time. The conversion rate confirmed a higher number of conversions with TEP (1.57%) than with TAPP (0.75%), consistent with Wake et al.’s findings, and concluded the lack of sufficient evidence to recommend the use of TEP rather than TAPP.

In 2019, Aiolfi et al. (7) wrote a Bayesian network meta-analysis that compared the open approach, TAPP, TEP, and r (robotic) TAPP and included 16 studies. The inclusion criteria were male patients who underwent surgical repair for a primary unilateral inguinal hernia. It gathered 17,112 patients for TAPP and 15,687 for TEP with postoperative follow-up ranging between 1 and 60 months. The primary outcome analyzed were hematoma formation (no statistical difference, RR 1.01; 95% CrI 0.51–1.80), seroma formation (no statistical difference, RR 0.70; 95% CrI 0.39–1.31), postoperative chronic pain (no statistical difference, RR 1.70; 95% CrI 0.63–3.20), and recurrence (no statistical difference, RR 1.10; 95% CrI 0.63–2.10). The secondary outcomes were SSI (no statistical difference, RR 0.90; 95% CrI 0.39–2.21, RR 1.10), urinary retention (no statistical difference, RR 1.10; 95% CrI 0.49–2.57), and operative time (no statistical difference, smd = −3.60; 95% CrI−7.70 to 0.58), concluding that there was no superiority of one technique over the other.

In 2020, a systematic review by Hung et al. (29) compared 659 patients in the TEP group to 682 patients in the TAPP group from 14 trials. The outcomes analyzed were seroma formation, edema, hematoma, intraoperative injury, urinary retention, epigastric vessel bleeding, and wound infection. The TEP group had a higher seroma rate than the TAPP group (Peto odds ratio = 2.01; 95% CI, 1.39–2.91), although TEP had a lower scrotal/cord edema rate at immediate postoperative (Peto odds ratio = 0.22; 95% CI, 0.09–0.57) and 1 week after inguinal hernia repair (Peto odds ratio = 0.58; 95% CI, 0.37–0.91) than TAPP. The other results showed no significant difference between the two techniques.

In 2021, Aiolfi et al. (30) wrote a systematic review that compared TEP and TAPP and included fifteen RCTs (1,359 patients), in which 702 (51.6%) underwent TAPP repair, and 657 (48.4%) underwent TEP repair. The age of the patients ranged from 18 to 92 years, and 87.9% were male. The estimated pooled RR for hernia recurrence (RR = 0.83; 95% CI 0.35–1.96) and chronic pain (RR = 1.51; 95% CI 0.54–4.22) were similar for TEP vs. TAPP. No significant differences were found in terms of early postoperative pain, operative time, wound-related complications, hospital length of stay, return to work/daily activities, and costs. In conclusion, the cumulative evidence and information size were sufficient to provide conclusive evidence on recurrence and chronic pain, and similar trials or meta-analyses seemed unlikely to show diverse results and should be discouraged.

An interesting review on the surgical management of the sportsman's hernia was written in 2021 by Kler et al. (31), specifically comparing TEP and TAPP in this different research field. The sportsman's hernia does not involve a true herniation and is more appropriately referred to as inguinal disruption. The review included a total of 28 reports, of which 22 were cohort studies, 4 were case series, and 2 were RCTs. A total of 1,473 patients underwent TAPP, and 715 underwent TEP. The outcomes analyzed were return to sporting activity (28 days with a range of 3 weeks to 3 months for both surgical modalities), total pain reduction after 3 months (94.0% when combining results of both modalities), and complications (1.8% for both modalities). No significant differences were found, confirming comparable efficacy and safety of the two techniques also in treating a different disease. Table 1 summarizes the outcomes evaluated by each systematic review, with the final evidence underlying how the two techniques are comparable, safe, and feasible in every setting.

**Table 1:** Literature summary on the TAPP and TEP comparison.

Author	No. of patients TAPP–TEP	Outcomes evaluated	Conclusions
Wake et al. (13)	11,651–7,043	Vascular injuries, visceral injuries, deep/mesh infections, port site hernias, conversion operation time, intraoperative complications, hematoma formation, time to return to usual activities, time to return to work, length of hospital stay, and recurrence at 3 months	Data not conclusive
Bracale et al. (14)	395–1,209	Operative time, postoperative pain, hospital stay, time to return to work, postoperative complications, and recurrences	No significant differences between the two techniques
Aiolfi et al. (4)	17,112–15,687	Primary outcomes: hematoma formation, seroma formation, postoperative chronic pain, recurrence; secondary outcomes: SSI, urinary retention, and operative time	No significant differences between the two techniques
Hung et al. (19)	682–659	Seroma formation, edema, hematoma, intraoperative injury, urinary retention, epigastric vessel bleeding, and wound infection	TEP higher seroma rate TEP lower scrotal edema
Aiolfi et al. (15)	702–657	Hernia recurrence, chronic pain, early postoperative pain, operative time, wound-related complications, hospital length of stay, return to work/daily activities, and costs	No significant differences between the two techniques
Kler et al. (16)	1,473–715 Sportsman hernia patients	Sporting activity, total pain reduction after 3 months, and complications	No significant differences between the two techniques

## Future perspectives

The literature review reveals that both procedures are comparable in direct and indirect analyses, suggesting that further comparative analysis or RCT is useless. At present, the robotic approach seems to be the only real news on inguinal hernia treatment, even useful as the training model for abdominal robotic surgery, with outcomes in the short term comparable to that of the laparoendoscopic approach. As reported by the recently published systematic review and meta-analysis by Solaini et al. (32) on 64,426 patients, chronic pain, postoperative complications, and conversion rate are similar between the two techniques. Costs and operative time (longer in a robotic group) remain a hot issue to be solved in the future.

## Conclusions

TAPP and TEP represent two standard laparoendoscopic procedures with over 30 years of history and practice. Personal preference and differences in education move the surgical choice between them, and the literature evidence does not gain the level of specific recommendations for one over the other approach. Personally, and based on published results, the hernia surgeon should manage both techniques and recommend operations based on personal expertise, hernia/patient characteristics, and locally available technology.
